# Unnecessary orchiectomy due to atypical sarcoidosis manifesting as a unilateral scrotal mass: a case report and literature review

**DOI:** 10.3389/fimmu.2023.1253120

**Published:** 2023-11-27

**Authors:** Valeria Skopelidou, Pavel Hurník, Vladimir Židlík, Lubomír Tulinský, Jiří Lenz, Tomáš Balner, Dušan Žiak, Patricie Delongová, Rudolf Karas, Miroslava Škripková, Matěj Jendřejek

**Affiliations:** ^1^ Institute of Molecular and Clinical Pathology and Medical Genetics, University Hospital Ostrava, Ostrava, Czechia; ^2^ Institute of Molecular and Clinical Pathology and Medical Genetics, Faculty of Medicine, University of Ostrava, Ostrava, Czechia; ^3^ Department of Pathology, EUC Laboratoře CGB a.s., Ostrava, Czechia; ^4^ Department of Surgery, University Hospital Ostrava, Ostrava, Czechia; ^5^ Department of Surgical Studies, Faculty of Medicine, University of Ostrava, Ostrava, Czechia; ^6^ Department of Pathology, Znojmo Hospital, Znojmo, Czechia; ^7^ Department of Allergology and Clinical Immunology, University Hospital Ostrava, Ostrava, Czechia; ^8^ Department of Radiology, Faculty of Medicine, University of Ostrava, Ostrava, Czechia; ^9^ Department of Imaging Methods, Faculty of Medicine, University of Ostrava, Ostrava, Czechia; ^10^ Department of Radiology, Palacký University Olomouc, Olomouc, Czechia; ^11^ Department of Pulmonary Diseases and Tuberculosis, University of Ostrava, Ostrava, Czechia

**Keywords:** sarcoidosis, testicular sarcoidosis, extrapulmonary sarcoidosis, genitourinary sarcoidosis, orchiectomy, case report

## Abstract

Sarcoidosis is a disease characterised primarily by lung tissue involvement. Extrapulmonary involvement, particularly in the genitourinary tract, is extremely rare, particularly when it comes to primary disease detection in this location. The gold standard in establishing a definitive diagnosis of sarcoidosis is a combination of the clinical picture, the results of imaging methods, and histopathological examination from the biopsy taken (thus ruling out other causes of granulomatous inflammation). However, it is common for the biopsy to be infeasible or for the patient to refuse such an examination, resulting in the neglect of this critical verification. We introduce the case of a young 29-year-old man of Czech nationality who had been complaining for some time about non-specific pain above the pubic bone and in the lower abdomen, which was combined with a painless enlargement of the right half of the scrotum. Due to suspected malignancy, it was, after considering clinical, imaging, and laboratory findings, decided to perform a radical orchiectomy as a treatment option. The histological examination revealed that it was not cancer, but rather a rare genitourinary form of extrapulmonary sarcoidosis. In this case, radical resection had been, therefore, unnecessary. We also present a review of the literature on published extrapulmonary, genitourinary, and testicular sarcoidosis cases. All the above demonstrates the importance of considering a possible atypical sarcoidosis manifestation and histological confirmation before pursuing radical solutions.

## 1 Introduction

Sarcoidosis is a chronic multisystemic granulomatous disease of unknown origin, which affects various organs and tissues, with the lungs and hilar lymph nodes being the most commonly impacted (approximately 90% of all cases). Multiple non-caseating granulomas formed mostly by an accumulation of fibroblasts, lymphocytes, and transformed macrophages are a typical morphological picture (1–4). The exact pathogenesis of sarcoidosis has not been completely elucidated yet. Recent advances in the understanding of the function of various immune cells in sarcoidosis suggest that an important role in granuloma formation and progression is played by Th1 cells, Th17 cells, Th17.1 cells, Treg cells and functional phenotypes of macrophages (5).

It is primarily a disease of younger and middle-aged individuals, most frequently between the ages of 20 and 40. The diagnostic process is usually lengthy and complicated since the disease’s symptoms are often non-specific and can mimic other illnesses (including malignancy), particularly in atypical locations. The testicular form of sarcoidosis is extremely rare (less than 50 cases had been reported in the literature since the year 2000). This rare condition, however, bears a high risk of performing an unnecessary orchiectomy and/or epididymectomy due to a suspicion of malignancy, especially where diagnostic biopsy is not performed (6–9).

**Figure 1:**
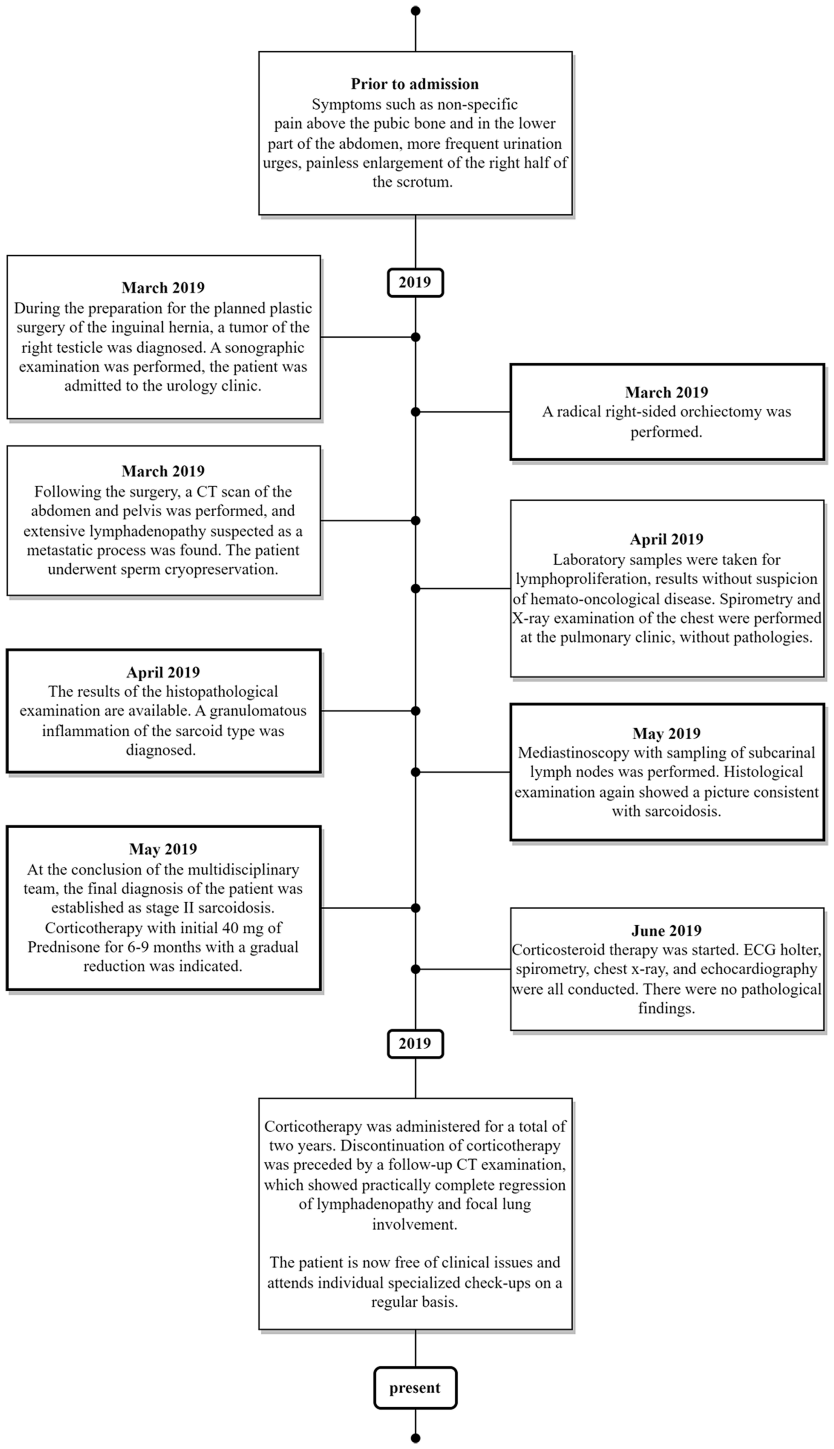
The timeline of the main diagnostic and therapeutic points of this case.

Here, we would like to introduce the case of a patient whose first sarcoidosis manifestation was a painless mass in the right half of the scrotum. To provide a more comprehensive view of this problem, this paper also includes a review of published literature on atypical extrapulmonary manifestation of sarcoidosis – testicular sarcoidosis. The primary objective of this publication is to emphasise that this disease should not be overlooked in the differential diagnosis and that preoperative biopsy can be extremely beneficial in certain cases. The case report itself was created using the CARE checklist (**Supplementary Material 1**). The timeline of the main diagnostic and therapeutic points of this case is depicted in **Figure 1**.

## 2 Case report

We present the case of a young 29-year-old man of Czech nationality referred for plastic surgery of a recurrent right inguinal hernia with scar excision. During the pre-operative check, however, an enlargement of the right testicle was discovered, bringing attention to the possibility of a testicular tumour for the first time. Following that, the patient was transferred to the urology clinic for a more thorough evaluation. It was discovered that the patient had non-specific pain above the pubic bone and in the lower part of the abdomen for an extended period of time. He also noticed more frequent urination urges and there was also a longer-term (more than three months) painless enlargement of the right half of the scrotum. The patient denied having any other problems, he had no loss of appetite or weight loss, and there had been no previous genital trauma that could have caused the enlargement. In the past, an orchidopexy was performed due to the undescended right testicle. The patient also underwent inguinal hernia surgery in his childhood. The patient had essential hypertension, which was controlled with medication. Clinically, the patient’s family history was insignificant. Other information that could explain the patient’s problems or be relevant to the given case has not been discovered.

**Figure 2:**
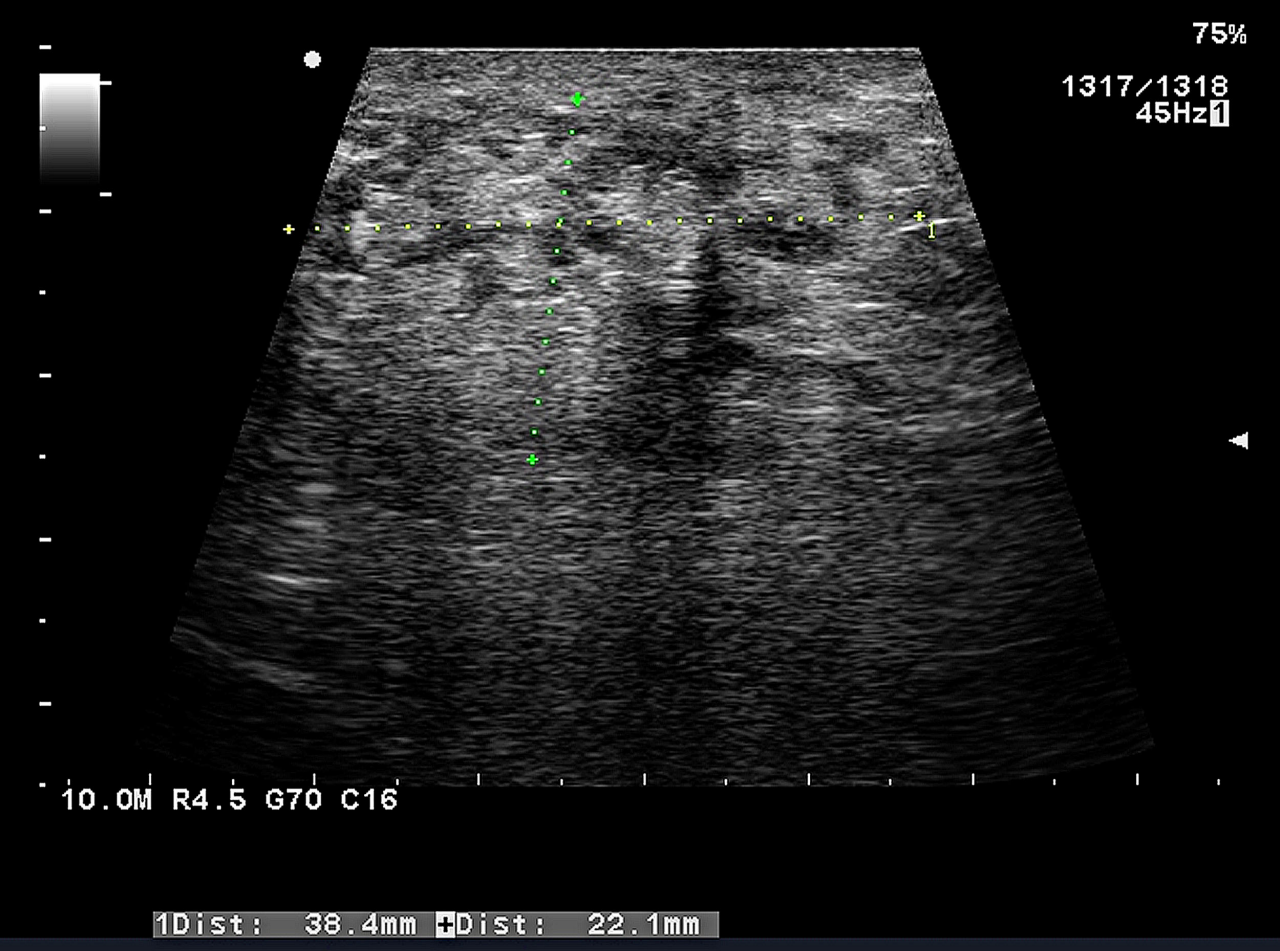
Ultrasound examination of the contents of the patient’s scrotum (in this case, the right testicle) performed prior to the final indication for surgery. According to the final evaluation, it was determined that it is a malignant tumour developing from testicular tissue that also extends into the epididymis, causing induration in this location. As previously noted in the text, the dilation of the veins of the pampiniformis plexus of the first degree on the left was a secondary finding. No additional pathologies were discovered. This was the decisive examination on the basis of which the patient was indicated for orchiectomy.

The patient’s physical clinical examination revealed a significantly hard testicle on the right, as well as an infiltrated and firmer spermatic cord. The findings on the left side were normal. Based on the results of the subsequent ultrasound examination, a diagnosis of a tumour arising from the testicular tissue with simultaneous induration (suspicious malignant infiltration) of the epididymis on the right was made (**Figure 2**). The left testicle had no changes in echogenicity and any focal pathologies, but dilatation of the veins of the pampiniform plexus was detected. Other findings were either normal or clinically insignificant in this case.

A radical right-sided orchiectomy was chosen as the solution. No preoperative or perioperative biopsy was performed since the diagnosis of malignancy was considered certain. The possible risk of malignant cells spreading and thus worsening the prognosis of a patient with the suspected testicular tumour was the main reason for the surgery. Given the seriousness of the presumed diagnosis, the quickest possible intervention was preferred and inguinal exploration with radical orchiectomy was performed.

**Figure 3:**
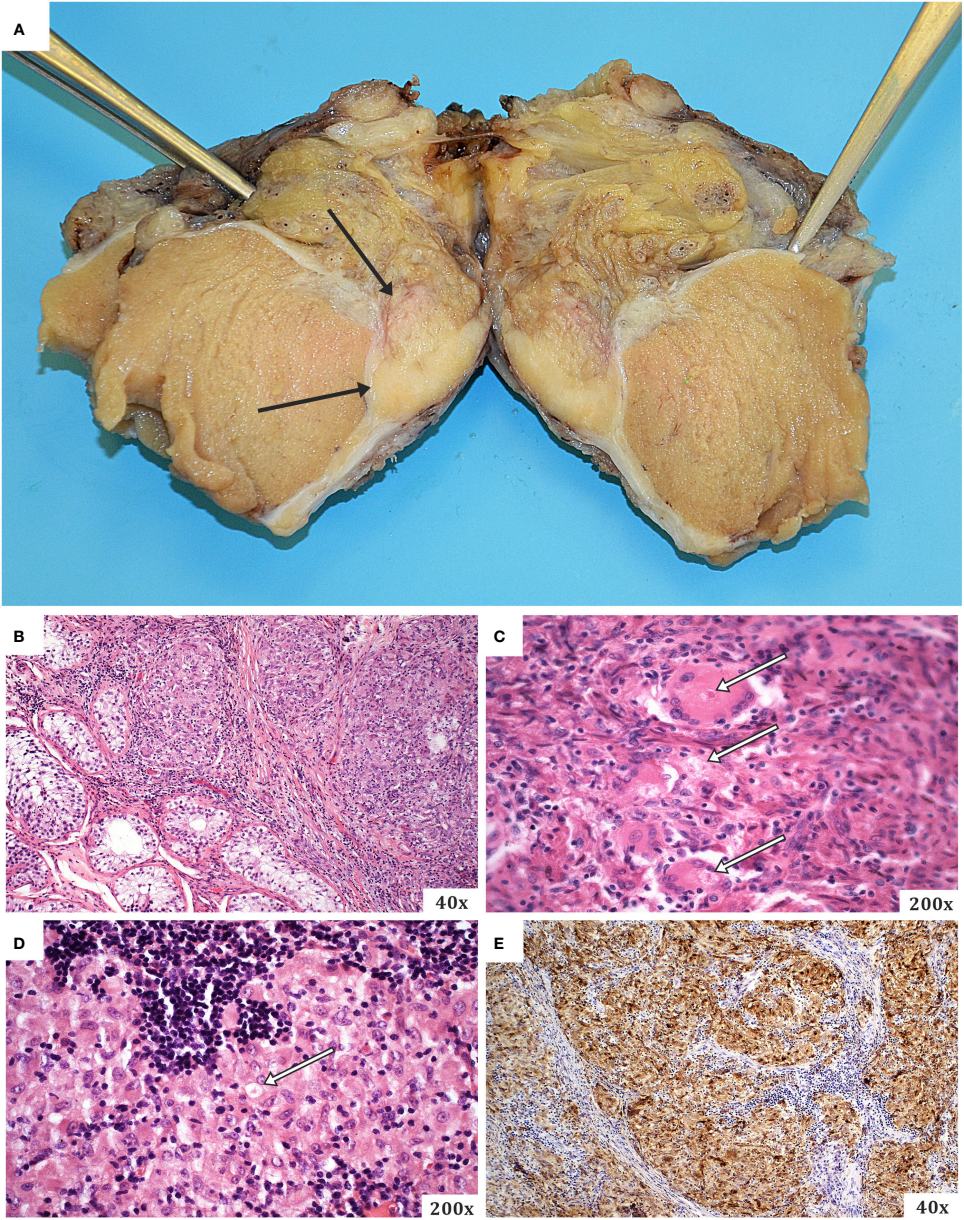
The testicle, epididymis, and spermatic cord complex **(A)** were sent to the Pathology department of the Ostrava University Hospital following a radical orchiectomy. The testes and spermatic cord showed no obvious macroscopical pathology. The testis sheaths were slightly thickened. However, a solid lesion approx. 1 × 2 × 2 cm in size primarily whitish in colour was present in the epididymis (black arrows on the left, the lesion can be seen on both sides of the cut). **(B–E)** demonstrate the typical microscopic structures seen in sarcoidosis when stained with haematoxylin and eosin, particularly confluent non-caseating epithelioid granulomas. Another common trait is the presence of numerous asteroid bodies (white arrows with black outlines). Rich lymphocytic infiltration can also be seen around the granulomas. **(E)** shows the immunohistochemical staining for CD68 (histiocytic marker), which was highly positive in our case, as is typical of this type of cells.

A complex of the testis, epididymis and spermatic cord (right side) with a total weight of 55 g and a size of 6.5 × 4 × 4 cm was sent to the Institute of Pathology for histological examination. The seminal cord was 1 cm long and had a normal macroscopic structure. The testicle was 3 × 3 × 3.5 cm in size, with light brown parenchyma and no macroscopically visible focal changes. The testis sheaths had been slightly thickened. The epididymis was 2.5 × 0.7 × 1 cm in size, and a well-circumscribed solid whitish lesion was visible macroscopically. There were no other significant macroscopic findings (**Figure 3**).

Microscopic histopathologic examination of all the mentioned structures did not confirm the initial clinical diagnosis of a malignant tumour. Non-caseating epithelioid cell granulomas with multinucleated giant cells were the most dominant finding in all specimens. Asteroid inclusions were also visible in the giant cells, as well as an optically active material with a foreign appearance and a morphology that was suggestively crystalline or fibrous. Granulomas were found to express CD68 diffusely using immunohistochemical techniques. CKAE1/AE3 expression, on the other hand, was not observed (**Figure 3**). In the differential diagnosis, sarcoidosis was the most likely finding due to the typical histopathological results.

Following the surgery, a full-body CT (computed tomography) scan revealed extensive lymphadenopathy, particularly in the mediastinal, paratracheal, and hilar nodes. The lung parenchyma also demonstrated focal involvement. Additionally, there were enlarged nodes in the supraclavicular sheath and retroperitoneum. Based on these findings, the clinicians suspected a progressive tumour process. The fact that the oncological markers were negative while the testicle was obviously affected with a pathological growth, led the clinicians to suspect lymphoma. A mediastinoscopy with subcarinal lymph nodes sampling for histopathological examination was performed; similar to the scrotal contents, histopathology revealed the presence of typical sarcoidosis signs.

Tumour markers were assessed on Day 3 day after the surgery. Both the human chorionic gonadotropin level and alpha fetoprotein level were within the normal range.

The laboratory evaluation on Day 10 after the orchiectomy showed physiological liver transaminases, alkaline phosphatase, lactate dehydrogenase, glutamyltrasferase, creatinine, urea, albumin, total serum protein, C-reactive protein and immunoglobulins (IgG, IgM, IgA) results. Serum protein electrophoresis showed a polyclonal increase in gamma globulin. Further laboratory testing showed a decreased haemoglobin level of 12.6 g/dl, elevated platelet count of 489 × 10^9^/l and lymphopenia with a level of lymphocytes 0.7 × 10^9^/l. Blood lymphocyte immunophenotyping by flow cytometry wasn´t performed.

As a result, the patient’s condition was concluded by the multidisciplinary team as stage II sarcoidosis. Specific examinations were also performed to detect associated eye and heart impairment. The findings (including serum calcium level)were normal, with the exception of a 24-hour urine collection test, showing increased calcium excretion and serum angiotensin-converting enzyme (SACE), which was below the normal range at 13 (reference range: 20-70). The patient was then prescribed corticosteroid therapy. Prednisone was administered at a dose of 40 mg/day per os for the first three weeks, then at a dose of 30 mg for two weeks, and finally at a dose of 20 mg long-term. Corticotherapy was administered for a total of two years. Discontinuation of corticotherapy was preceded by a follow-up CT examination, which showed practically complete regression of lymphadenopathy and focal lung involvement.

The patient is now free of clinical issues and attends individual specialised follow-ups on a regular basis, with the last one having taken place in June of this year (2023).

## 3 Discussion

Sarcoidosis, as mentioned in the Introduction, is a disease characterised mostly by the involvement of lung tissue and related lymph nodes (mainly hilar). Only approximately one-tenth of all sarcoidosis cases primarily manifest as extrapulmonary (i.e., without any pulmonary lesion). Extrapulmonary manifestations primarily occur in the skin, eyes, spleen, liver, or, in some cases, the central nervous system. Primary sarcoidosis in the genitourinary tract is extremely rare, especially when it comes to the primary detection of the disease in this location. As a consequence, sarcoidosis is rarely considered as a possible cause of pathological findings in these unusual locations, putting patients at risk of unnecessary procedures and surgical interventions that can negatively impact their quality of life (3, 4, 8, 10, 11).

The genitourinary manifestation of sarcoidosis affects less than 0.2% of all patients. It’s worth noting that this statistic increases to 5% when autopsies are included – genitourinary involvement is frequently underdiagnosed (7, 12, 13). A review of the literature, including a total of 60 case reports of sarcoidosis affecting the male reproductive system, was published in 2004 (14). According to their findings, the epididymis (73%) is the most commonly affected, followed by the involvement of testicles (47%), spermatic cord (8%), and prostate (3%). In addition, we conducted a literature review, which included published case reports from 2004 to the present and found 35 publications that matched our search criteria (**Table 1**). Using the keywords “extrapulmonary sarcoidosis,” “genitourinary sarcoidosis,” and “testicular sarcoidosis,” a total of 598 articles were found in the PubMed database. The search criteria were publications with full text available from 2004 to the present day. Individual abstracts that were irrelevant to the topic were excluded after a thorough review. Duplicate articles or those lacking histopathological verification or other critical data were also excluded. As a result, 35 articles regarding male reproductive tract sarcoidosis (excluding prostate – 1 case) were included in the final analysis. **Table 1** shows the results of the search. According to the findings of the presented sources, the testicles were the most commonly affected (71%), followed by the epididymis (43%) and, finally, the spermatic cord (6%). It was usually a bilateral involvement (57%). The vast majority of presented cases had the primary manifestation in this atypical localization (86%), only in a few cases (14%) did the patient have a known clinical history of sarcoidosis. In roughly half of the cases (54%), no preoperative verifying biopsy or frozen biopsy during exploratory surgery was performed, which partly contributed to the fact that in 11 cases (31%), a radical procedure (orchiectomy or epididymectomy) was performed. Another interesting finding was the fact that simultaneous involvement of the testis, epididymis, and spermatic cord similar to our patient was described only in a single report; in that case, however, the patient had already been previously diagnosed with sarcoidosis. In effect, the case report we present is unique in its simultaneous unilateral involvement of all the mentioned structures representing the first manifestation of sarcoidosis.

**Table 1:** Review of testicular sarcoidosis cases reported since the year 2004.

Case	Year	Country	Age/Sex	Initial presentation	Primarily affected organ	Prior diagnosis	Imaging methods used	Testicular biopsy	Histological features	Treatment	Outcome	Ref
1	2022	Poland	33/M	enlarged mass in the left groin	spermatic cord, epididymis, bilateral hilar nodes	no	US, CT, X-ray	yes	noncaseating granulomas	surgery (spermatic cord excision, intraoperative examination)	2 m: regression	Ostrowska et al. (6)
2	2022	Turkey	23/M	left testicular pain	hilar nodes, newly testes	yes	US, CT, MRI	yes	noncaseating granulomas	Corticosteroids	6 m: regression	Haciosmanoglu et al.
3	2022	Portugal	33/M	post-coital scrotal pain, pain during scrotal palpation, dry cough	testes, liver, spleen	no	US, CT	no (only liver biopsy)	noncaseating granulomas	–	24 m: no new symptoms	Campos et al. (11)
4	2022	USA	40/M	painless scrotal mass	testes, epididymises, skin	no	US, X-ray, CT	no (only skin biopsy)	noncaseating granulomas	–	–	Choksi et al. (12)
5	2021	Belgium	32/M	painful swelling in the right hemiscrotum	epididymises, testis, bilateral hilar nodes	no	US, CT	no (only lymph nodes)	noncaseating granulomas	surgery (scrotal exploration by inguinal approach), Corticosteroids	8 m: regression, genital tract potency was not restored	Ballet et al. (7)
6	2021	Italy	42/M	upper left quadrant pain	testes, multiorgan involvement	no	US, CT, PET/CT	yes	noncaseating granulomas	Corticosteroids, Methotrexate	12 m: regression	Cinque et al. (15)
7	2020	Turkey	31/M	infertility, cough	testes, lungs	no	US, MRI, X-ray, CT	yes	noncaseating granulomas	Corticosteroids	12 m: regression	Albayrak et al. (1)
8	2020	Morocco	61/M	right-sided testicular pain, scrotal enlargement	testis	no	US, CT	no	noncaseating granulomas	surgery (radical right orchiectomy)	4 m: regression	Bouytse et al. (16)
9	2020	India	66/M	fever, weakness	testis, hilar nodes	no	PET/CT, US	no (only bone marrow)	noncaseating granulomas	surgery (left high inguinal orchiectomy)	–	Parida et al. (17)
10	2020	India	42/M	cough, weight loss, hyporexia	testes, multiorgan involvement	no	X-ray, CT, MRI	no (only cervical node biopsy)	noncaseating granulomas	surgery (high inguinal orchiectomy), Corticosteroids	12 m: regression	Bala et al. (18)
11	2019	Japan	26/M	painless bilateral scrotal mass	testes, epididymises, hilar nodes	no	US, X-ray, CT	yes	noncaseating granulomas	–	7 m: regression	Konishi et al. (19)
12	2019	Japan	32/M	painless bilateral scrotal swelling	lungs, newly testes	yes	US, CT, MRI, scintigraphy	yes	noncaseating granulomas	Corticosteroids	7 m: regression	Kimura et al. (10)
13	2019	UK	25/M	right-sided testicular pain	testes, multiorgan involvement	no	US, CT	yes	noncaseating granulomas	surgery (exploration of right testicle, excision)	? m: asymptomatic, no steroid therapy yet	Chierigo et al. (20)
14	2018	Switzerland	29/M	lumps in the right testis	testes, lungs	no	US, CT	yes	noncaseating granulomas	–	–	Babst et al. (21)
15	2015	USA	40/M	painful bilateral scrotal swelling	testes, multiorgan involvement	no	US, CT, X-ray	no	noncaseating granulomas	surgery (partial orchiectomy), Corticosteroids	? m: regression	Patel et al. (22)
16	2014	UK	27/M	progressive left-sided testicular and epididymal swelling	hilar lymph node, newly testis	yes	US	no (only lymph node)	noncaseating granulomas	anti-tuberculosis medication, surgery (inguinal orchiectomy), Corticosteroids	24 m: regression	Joel et al. (9)
17	2013	USA	33/M	right upper chest pain, non-productive cough, weight loss, right testicular mass	epididymis, testis, lungs	no	US, CT	no	noncaseating granulomas	surgery (radical orchiectomy), Corticosteroids	? m: regression	Esnakula et al. (4)
18	2013	USA	37/M	bilateral leg weakness, headaches, urinary retention, impotence	testis, CNS	no	MRI, CT, US	yes	necrotizing and noncaseating granulomas	Corticosteroids	3 m: regression	Alraies et al. (23)
19	2012	Canada	37/M	infertility	testes, hilar nodes, lungs	no	X-ray, CT	yes	noncaseating granulomas	Corticosteroids	? m: regression	Kovac et al. (24)
20	2011	Belgium	40/M	painless mass of the right testis, dry cough	testis, multiorgan involvement	no	US, X-ray, CT	no (only lymph node)	noncaseating granulomas	Corticosteroids	–	Eyselbergs et al. (3)
21	2011	Turkey	25/M	right scrotum stiffness, left scrotal pain and tenderness	hilar nodes, newly epididymises	yes	MRI	yes	noncaseating granulomas	surgery (scrotal exploration), Corticosteroids	? m: regression	Canguven et al. (25)
22	2011	USA	30/M	frontal headache, skin nodules, painless right hemiscrotal mass	epididymises, multiorgan involvement	no	CT, US	no	noncaseating granulomas	surgery (right orchiectomy), Corticosteroids	3 m: regression	Gupta et al. (26)
23	2011	South Korea	27/M	palpable testicular mass	testis, multiorgan involvement	no	US, X-ray, CT	yes	noncaseating granulomas	surgery (inguinal exploration), Corticosteroids	4 m: regression	Kim et al. (27)
24	2010	UK	50/M	lethargy, weight loss, palpable scrotal mass	hilar nodes, newly multiorgan involvement, testis, epididymis, spermatic cord	yes	US, CT	no	noncaseating granulomas	surgery (left orchiectomy), Corticosteroids	24 m: regression	Woolf et al. (28)
25	2009	USA	39/M	asymptomatic right-sided epididymal mass	epididymis	no	US, CT	no	noncaseating granulomas	surgery (exploration, excision), NSAID	–	Hey et al. (2)
26	2009	Egypt	29/M	infertility	epididymises, hilar and subcarinal nodes	no	US, MRI, X-ray, CT	yes	noncaseating granulomas	surgery (epididymal exploration), Corticosteroids	3 m: regression, azoospermia remained	Hassan et al. (29)
27	2008	France	31/M	painful right testicular enlargement	testis	no	US, CT	no	noncaseating granulomas	surgery (right orchiectomy)	24 m: regression	Thuret et al. (13)
28	2007	UK	38/M	bilateral testicular swelling, night sweats, weight loss	testes	no	US, X-ray, CT	yes	noncaseating granulomas	anti-tuberculosis medication, Corticosteroids	36 m: regression, oligospermia	Datta et al. (30)
29	2007	Japan	31/M	painless scrotal swelling	epididymis	no	US, X-ray	no	noncaseating granulomas	surgery (scrotal exploration, epididymectomy, excision of nodules), Corticosteroids	6 m: regression	Obinata et al. (31)
30	2006	USA	24/M	bilateral testicular pain and swelling, weight loss	testes, epididymis, multiorgan involvement	no	US, CT	yes	noncaseating granulomas	surgery (scrotal exploration, excision of lesions)	–	Massarweh et al. (32)
31	2006	USA	29/M	painful scrotal swelling	scrotum, epididymis, multiorgan involvement	no	US, CT, MRI	yes	noncaseating granulomas	Corticosteroids	6 m: regression	Vahid et al. (33)
32	2005	USA	29/M	right testicular pain, pleuritic chest pain	testes, epididymises, multiorgan involvement	no	US, X-ray	yes	noncaseating granulomas	surgery (high inguinal exploration)	–	Rehman et al. (34)
33	2004	UK	35/M	painless scrotal swelling, submental mass, skin lesions	testes, skin, hilar nodes	no	US, CT	no (only from other masses)	noncaseating granulomas	Corticosteroids	? m: regression	Khan et al. (35)
34	2004	Japan	46/M	painless bilateral scrotal swelling	epididymises, skin	no	US, MRI, X-ray, CT	no	noncaseating granulomas	surgery (bilateral epididymectomy)	–	Kodama et al. (14)
35	2004	UK	27/M	painless mass in right testis	testis	no	US	yes	noncaseating granulomas	Corticosteroids	7 m: regression	Rees et al. (36)

CNS, central nervous system; CT, computed tomography; F, female; M, male; MRI, magnetic resonance imaging; NSAID, non-steroidal anti-inflammatory drugs; PET/CT, positron emission tomography/computed tomography; US, ultrasound.

Clinical symptoms of testicular involvement are numerous and differ from case to case. It can be a sharp pain that appears suddenly or a long-term silent mass in the scrotum (as in our case). However, it is necessary not to forget that the scrotal enlargement can be caused by a wide range of diseases that must be ruled out before a definitive diagnosis can be made. It may be a hernia, an abscess, a localised form of tuberculosis or syphilis, or even a malignant tumour (a primary or secondary site, or even a manifestation of lymphoma). Sarcoidosis is typically diagnosed by exclusion (6, 11, 21, 28, 29).

A combination of the clinical picture, the results of imaging methods, and the conclusion of histopathological examination from the biopsy taken (thus also ruling out other causes of granulomatous inflammation) is the gold standard in establishing a definitive diagnosis of sarcoidosis. However, this is a procedure reserved for cases of lung tissue or lymph node abnormality. Exact guidelines for cases of primary manifestation in unusual locations are still lacking. Moreover, biopsy is relatively often not completely feasible or the patient refuses such examination, resulting in the omission of this critical verification. In such cases, the final decision is based solely on clinical examination and imaging modalities, which do not provide an accurate picture of the disease at hand. Sarcoidosis can also mimic other lesions, the most serious of which is a malignant tumour, which cannot be ruled out without a confirmative biopsy (1, 3, 6, 7, 19, 20).

Malignancy is the most serious possible diagnosis, which is further complicated by the fact that both sarcoidosis and malignant testicular tumours have a similar age of onset. Even though histopathological evaluation is the most sensitive and accurate method, clinical practice relies largely on imaging results, particularly on the ultrasound, which was also performed in our case. It can reliably identify a pathological lesion, but it is unable to distinguish between “mere” extrapulmonary sarcoidosis and a malignant tumour. The use of MR (magnetic resonance) imaging is an option, but it is a much more expensive examination with several limiting criteria, and the data obtained may not be strictly specific (3, 6, 9, 15). Furthermore, certain serum markers associated with testicular tumours – LDH (lactate dehydrogenase), AFP (alpha-fetoprotein), HCG (human chorionic gonadotropin), and ACE (angiotensin-converting enzyme) – are evaluated in the laboratory. However, even in the case of benign lesions, their values can be false positive. At the same time, if these oncomarkers are negative, a malignant lymphoma differential diagnosis is still not ruled out (3, 7, 11, 16, 18).

As a result, it is appropriate to highlight the indispensable role of histopathological examination, which can determine the final diagnosis with high accuracy. Even so, since biopsy is an invasive method that is associated with several complications in the genital area, this examination is performed less frequently and reluctantly. When a malignant tumour is suspected, a large number of clinicians consider performing a probatory biopsy very carefully because there is a risk of spreading malignant cells to other locations. It is also worth noting that some studies suggest a link between testicular sarcoidosis and testicular cancer, but this remains a contentious issue. That is why we continue to face the fact that when a unilateral scrotal mass is discovered, the most common treatment is an often-unnecessary radical orchiectomy, as was the scenario in our case (2, 7, 9, 19, 20, 22, 28, 32, 35, 36).

As a result, some recommendations can be summarized in the following points. In younger individuals with a bilateral scrotal mass involving several scrotal structures, a known family or personal history of sarcoidosis and negative oncological markers, we should first consider a possible benign nature of the lesion and proceed with further verification. First and foremost, high-quality imaging of the chest, abdomen, and pelvis should be performed, and a biopsy with histological evaluation or perioperative biopsy should be seriously considered. Perioperative biopsy with immediate fresh-frozen section evaluation can be performed even if the clinical picture is unclear and the risk of malignancy is intermediate. This will allow, if the malignancy is indeed confirmed, to proceed with the radical procedure immediately while minimizing the risk of spreading of potential malignant cells; on the other hand, this approach will prevent unnecessary orchiectomy if the finding is benign (2, 3, 6, 7, 9, 10, 13, 14, 16, 19, 20, 28, 30, 32, 35). Still, if the clinical picture strongly indicates malignancy, biopsy is unnecessary and a radical solution should be employed.

However, it is clear that this is an issue that requires more research and a deeper understanding by professional societies in order to effect change and create precisely defined diagnostic and therapeutic procedures.

## 4 Conclusion

To summarise, the diagnosis of sarcoidosis, particularly when it comes to atypical localizations, remains a very complicated process, owing to the variability and non-specificity of the syndromes. Genitourinary sarcoidosis is a very rare manifestation of this systemic disease, and it frequently mimics other diseases (including cancer), making the correct diagnosis difficult. Hence, when an atypical scrotal mass is discovered, physicians should consider the atypical manifestation of sarcoidosis among the possible diagnoses. The presented case shows the importance of chest examination using imaging methods and of histopathological confirmation of the diagnosis in such unclear cases, in which sarcoidosis can be suspected, before performing a radical surgical procedure.

## 5 Patient perspective

The patient agreed to the course of treatment and had no objections to the chosen procedures. The patient also consented to the publication of his case with anonymized data, as he believes that this may help to raise awareness of the issue.

## Data availability statement

The original contributions presented in the study are included in the article/**Supplementary Material**. Further inquiries can be directed to the corresponding author.

## Ethics statement

The studies involving humans were approved by The Ethics Committee of FN Ostava, University Hospital Ostrava, 17. listopadu 1790/5, 708 52 Ostrava, Czech Republic (Reference number 430/2023). The studies were conducted in accordance with the local legislation and institutional requirements. The participants provided their written informed consent to participate in this study. Written informed consent was obtained from the individual(s) for the publication of any potentially identifiable images or data included in this article.

## Author contributions

All authors listed have made a substantial, direct and intellectual contribution to the work, and approved it for publication.
