# Supplementary figures and images for: Unnecessary orchiectomy due to atypical sarcoidosis manifesting as a unilateral scrotal mass: a case report and literature review
Source: Front Immunol. 2023 Nov 27;14:1253120. doi: 10.3389/fimmu.2023.1253120 (PMC10711076; doi:10.3389/fimmu.2023.1253120)

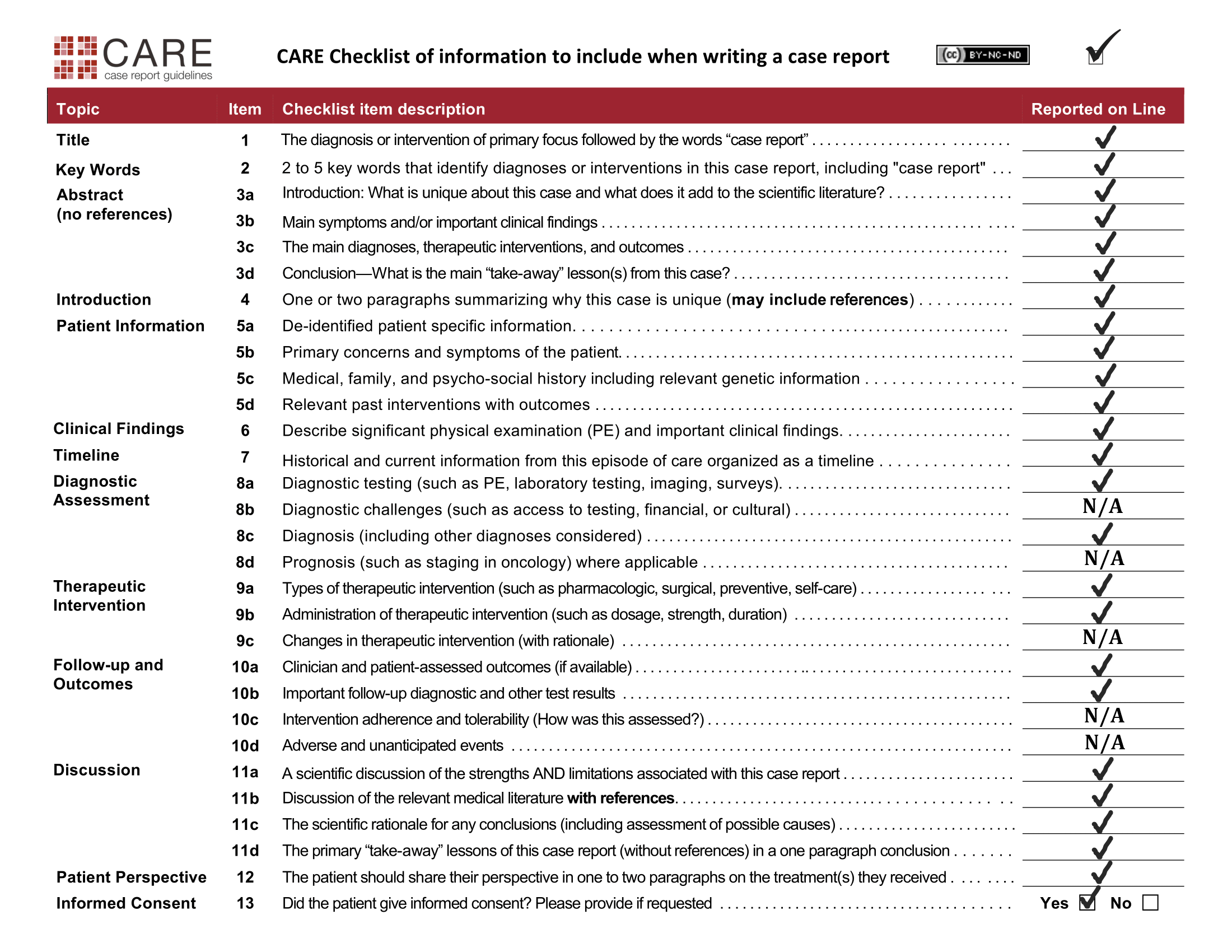

Supplement: Supplementary file 1 [file Image_1.png]
